# Pharmacological targeting of C3aR modulates mesangial matrix deposition in db/db mice

**DOI:** 10.7717/peerj.21248

**Published:** 2026-04-28

**Authors:** Jing Zheng, Xiaoting Wu, Mengjie Weng, Jianxin Wan, Xiaohong Zhang

**Affiliations:** 1 Department of Nephrology, Blood Purification Research Center, The First Affiliated Hospital, Fujian Medical University, Fuzhou, Fujian, China; 2 Department of Nephrology, National Regional Medical Center, Binhai Campus of the First Affiliated Hospital, Fujian Medical University, Fuzhou, Fujian, China; 3 Fujian Clinical Research Center for Metabolic Chronic Kidney Disease, The First Affiliated Hospital, Fujian Medical University, Fuzhou, Fujian, China

**Keywords:** Complement, C3a receptor antagonist, Diabetic kidney disease, Mesangial matrix

## Abstract

Complement activation significantly contributes to the progression of diabetic kidney disease (DKD). Recent studies have shown that renal levels of the complement component 3 (C3) and its receptor (C3aR) increase with disease progression. Mesangial matrix deposition in the glomeruli is an early pathological hallmark of DKD, but the potential role of C3aR in this process remains unclear. In this study, we used C57BLKS/JGpt wild-type (WT) mice, C57BLKS/JGpt db/db mice (a well-established model of type 2 diabetic nephropathy), and a pharmacological C3aR antagonist (C3aRA, SB290157 trifluoroacetate) to investigate this association. Strong C3 and C3aR staining was observed in the glomerular tufts of db/db mice, with C3aR co-localizing with mesangial nuclear cell markers. After 8 weeks of C3aRA treatment, db/db mice showed significant improvements in urine albumin-to-creatinine ratio (UACR), glomerular hypertrophy, and mesangial expansion. Inhibition of C3aR suppressed the expression of fibronectin (FN), osteopontin (OPN), and alpha-smooth muscle actin (α-SMA), indicating reduced mesangial matrix deposition. C3aRA also decreased renal deposition of calcineurin (CaN) and nuclear factor of activated T-cells 1 (NFATc1). These findings indicate a potential association between C3aR signaling and phenotypic and functional alterations in mesangial cells, which may be linked to the CaN/NFATc1 pathway. Given the well-documented pharmacological limitations of SB290157, our results do not confirm a definitive regulatory role for C3aR, but identify C3aR as a candidate mediator of mesangial matrix deposition in DKD, providing preliminary evidence that targeting C3aR may represent a potential therapeutic strategy for diabetic kidney disease.

## Introduction

The prevalence of diabetes mellitus (DM) is rapidly rising. According to the International Diabetes Federation, 10.5% of adults worldwide (537 million people) had diabetes in 2021, a figure projected to increase to 12.2% (783 million) by 2045, resulting in a substantial global healthcare burden (Sun et al., 2022). Diabetic kidney disease (DKD) is the most common and severe microvascular complication of DM (Selby & Taal, 2020). Among its earliest pathological changes is the deposition of the glomerular mesangial matrix. Increasing evidence indicates that DKD progression is closely linked to dysregulated complement activation within the innate immune system (Flyvbjerg, 2017). Complement C3 cleavage produces the active fragment C3a, which binds to C3aR on renal cells and contributes to glomerular and tubular injury (Gao, Cui & Zhao, 2020). For example, the C3aR antagonist SB290157 reduces renin-induced C3aR expression, ameliorates renin-dependent hypertension-induced tubulointerstitial fibrosis, and inhibits profibrotic tubular transformation (Wang et al., 2023).

Studies have shown that renal expression of C3a and C3aR increases in parallel with DKD progression and correlates positively with pathological indicators of kidney injury (Li et al., 2019a). Microarray analyses in C3aR knockout mice further suggest that C3a/C3aR signaling modulates macrophage cytokine secretion, thereby influencing inflammatory responses on T-cell adaptive immunity (Li et al., 2019b). In addition, C3aR antagonist improves podocyte mitochondrial function in BTBR ob/ob mice, underscoring the therapeutic potential of targeting C3aR in DKD.

Mesangial cells can transform into a myofibroblast-like phenotype (Zhang et al., 2012), characterized by hyperproliferation and increased production of extracellular matrix (ECM) proteins and modifying enzymes (Chung et al., 2020). Importantly, activation of C3a/C3aR signaling has been shown to increase ECM protein expression in carcinoma-associated fibroblasts (CAFs) (Shu et al., 2020). Because mesangial cells share functional properties with fibroblasts—particularly their ability to synthesize and secrete large amounts of ECM—this mechanism is highly relevant to mesangial matrix expansion in DKD (Thomas & Ford Versypt, 2022).

The calcineurin-nuclear factor of activated T-cells 1 (CaN/NFATc1) pathway plays a central role in mesangial cell hypertrophy, functional activation, and glomerulosclerosis (Gooch et al., 2001; Sugimoto et al., 2001). Inhibition of NFAT2 has been shown to reduce albuminuria and attenuate mesangial matrix expansion in db/db mice (Zhang et al., 2013). Together, these findings suggest that C3aR signaling may drive mesangial cell dysfunction and ECM accumulation, in part through CaN/NFATc1 activation.

In this study, we investigated the role of C3aR in mesangial matrix deposition using db/db mice treated with a C3aR antagonist (C3aRA). We evaluated mesangial cell phenotypic changes by measuring alpha-smooth muscle actin (α-SMA), a marker of myofibroblast-like transformation, as well as the expression of ECM proteins fibronectin (FN) and osteopontin (OPN). We also assessed changes in CaN/NFATc1 signaling. This work aims to clarify the role and mechanism of C3aR in mesangial matrix deposition and provide a new theoretical basis for developing therapeutic strategies for DKD.

## Materials and Methods

### Animal model

Eight-week-old male C57BLKS/JGpt db/db mice (*n* = 16; body weight 38–44 g) were used as a model for type 2 DM. Age- and sex-matched male C57BLKS/JGpt wild-type (WT) mice (*n* = 8; body weight 22–26 g), with the same genetic background, served as controls. All mice were procured from GemPharmatech Co., Ltd., Jiangsu, China (Animal Production License No. SYXK 2018-0008).

Animals were housed in the Experimental Animal Center of Fujian Medical University under specific pathogen-free conditions (22 ± 2 °C; 45–75% humidity; 12-h light/dark cycle). Two to three mice were kept per cage with free access to water and food, and bedding was changed daily. A 1-week acclimatization period preceded all experiments.

Mice were randomly assigned to either the db/db + vehicle group or the db/db + C3aRA antagonist (db/db + C3aRA) group using computer-generated randomization. The C3aRA compound (SB290157 trifluoroacetate; HY-101502A; MedChemExpress, Monmouth Junction, NJ, USA) was dissolved in DMSO and diluted with 40% PEG300, 5% Tween-80, and 53% normal saline to a final concentration of 2 mg/mL. The vehicle consisted of the same solution without SB290157. Both vehicle and C3aRA were administered intraperitoneally at 10 mg/kg every other day for 8 weeks at a fixed time.

At 17 weeks of age, mice were fasted for 24 h with free access to water. They were placed in metabolic cages for urine collection, then euthanized with an overdose of sodium pentobarbital (2% solution, 100 mg/kg, intraperitoneally). Blood and kidney tissue samples were collected. Humane endpoints were established according to the NIH Guide for the Care and Use of Laboratory Animals; none of the animals met these criteria, and all remained healthy throughout the study. All procedures were approved by the Fujian Medical University Institutional Animal Care and Use Committee (No: FJMU IACUC 2020-0105). The study followed ARRIVE 2.0 guidelines.

### Metabolic and biochemical analyses

Mice were housed individually in metabolic cages and fasted overnight with free access to water. Before euthanasia, 24-h urine samples were collected and their volume recorded. Samples were centrifuged at 1,500 rpm for 15 min at 4 °C, and the supernatant was aliquoted and stored at −80 °C for albumin and creatinine assays.

Blood was collected *via* terminal cardiac puncture under anesthesia. Serum was obtained by centrifugation at 3,000 rpm for 10 min at 4 °C, aliquoted, and stored at −80 °C. Serum glucose, total cholesterol, triglycerides, blood urea nitrogen, serum creatinine, and urinary albumin-to-creatinine ratio (UACR) were measured using standardized commercial assays in the Department of Laboratory Medicine, First Affiliated Hospital of Fujian Medical University, Fuzhou, China.

### Renal histopathology

Kidneys were fixed in 10% neutral buffered formalin, paraffin-embedded, and sectioned at 3 μm. Sections were deparaffinized, rehydrated, and stained with hematoxylin-eosin (H&E) staining, periodic acid-Schiff (PAS), and periodic acid-silver methenamine (PASM). Pathological changes were examined microscopically and documented photographically.

For mesangial expansion assessment, 20 non-sclerotic glomeruli per kidney were randomly selected from cortical regions after PAS staining. Images were acquired at 40× magnification using a Nikon Eclipse Ni-U microscope with a DS-Ri2 camera. All analyses were blinded and performed using ImageJ software (version 1.53k, National Institutes of Health, Bethesda, MD, USA).

The glomerular area was measured by manually outlining boundaries with the “Freehand Selection” tool. Mesangial matrix regions were quantified using the “Color Deconvolution” plugin with standardized thresholding. Mesangial expansion was expressed as the percentage of the glomerular area that was positive for PAS staining. Analyses were performed on 15–20 glomeruli per mouse (*n* = 8 per group) using Image-Pro Plus software.

### Enzyme-linked immunosorbent assay (ELISA)

Serum samples were thawed and centrifuged at 3,000 rpm for 5 min at 4 °C to remove particulates, then diluted 1:500 in 0.01 mol/L phosphate-buffered saline (PBS; pH 7.0–7.2). Circulating complement component 3a (C3a) levels were quantified using a commercial sandwich ELISA kit (Catalog #SEA387Mu; Cloud-Clone Corp., Wuhan, China) according to the manufacturer’s instructions. Absorbance was measured at 450 with a 570 nm reference wavelength using a SpectraMax M5 microplate reader (Molecular Devices, San Jose, CA, USA). Concentrations (ng/mL) were calculated by interpolation from a four-parameter logistic standard curve in SoftMax Pro v7.0 software. All samples were assayed in duplicate.

### Immunohistochemistry and quantitative analysis

Kidney sections (4 μm) were deparaffinized in xylene, rehydrated through graded ethanol, and immersed in distilled water. Antigen retrieval was performed in 0.01 M citrate buffer (pH 6.0) using microwave heating (700 W for 5 min, followed by 300 W for 10 min). After cooling to room temperature, the sections were washed with PBS (pH 7.4), blocked with 3% bovine serum albumin (BSA) for 1 h at 37 °C, and then incubated with primary antibodies at room temperature for 1 h. The following antibodies were used: Complement C3 (1:500, sc-28294; Santa Cruz Biotechnology, Dallas, TX, USA), C3aR (1:5,000, sc-133172; Santa Cruz Biotechnology, Dallas, TX, USA), Fibronectin (1:200, ab24130; Abcam, Cambridge, UK), Osteopontin (1:1,000, sc-21742; Santa Cruz Biotechnology, Dallas, TX, USA), a-SMA (1:200, YM3365; Immunoway Biotechnology, Plano, TX, USA) Calcineurin (1:2,000, sc-17808; Santa Cruz Biotechnology, Dallas, TX, USA), and NFATc1 (1:500, sc-7294, clone 7A6; Santa Cruz Biotechnology, Dallas, TX, USA). Antigen-antibody complexes were visualized with an Enhanced Polymer HRP Detection System (PV-9000; ZSGB-BIO, Beijing, China) and 3,3′-diaminobenzidine (DAB, ZLI-9017; ZSGB-BIO, Beijing, China) as the chromogen, producing brown staining. Slides were counterstained with Mayer’s hematoxylin, dehydrated, cleared in xylene, and mounted with neutral balsam.

Digital whole-slide images were acquired at 40× magnification with a Nikon Eclipse E100 microscope and DS-Ri2 camera. For each kidney, 15–20 glomeruli from cortical regions were randomly selected, excluding structurally compromised glomeruli. Semi-automated quantification of C3, C3aR, FN, OPN, and α-SMA expression (percentage of positive area) was performed using ImageJ software. Quantification involved manual tracing of glomeruli, Ruifrok-Johnston color deconvolution, optimized thresholding, and pixel-based area calculation.

### Immunofluorescence

Kidney cryosections (5 μm) were washed with PBS and permeabilized with 0.3% Triton X-100 (Sigma-Aldrich, St. Louis, MO, USA) for 15 min at room temperature (RT). Sections were blocked with 1% BSA in PBS for 30–60 min, then incubated overnight at 4 °C with paired primary antibodies diluted in 1% BSA/PBS: (i) mouse anti-C3 (1:100, sc-28294; Santa Cruz Biotechnology, Dallas, TX, USA) with rabbit anti-GATA3 (dilution 1:300, ab199428; Abcam, Cambridge, UK); and (ii) mouse anti-C3aR (dilution 1:200, sc-133172; Santa Cruz Biotechnology, Dallas, TX, USA) with rabbit anti-GATA3 (1:300; Abcam, Cambridge, UK).

After PBS washes, sections were incubated for 1 h at room temperature in the dark with Alexa Fluor 594-conjugated goat anti-mouse IgG (1:500, A-11032; Invitrogen, Carlsbad, CA, USA) and Alexa Fluor 488-conjugated goat anti-rabbit IgG (1:500, A-11008; Invitrogen, Carlsbad, CA, USA). Nuclei were counterstained with DAPI (5 μg/mL, D9542; Sigma-Aldrich, St. Louis, MO, USA). Samples were mounted in ProLong Gold Antifade Mountant (P36930; Invitrogen, Carlsbad, CA, USA) and imaged with a Zeiss LSM 880 confocal microscope (Oberkochen, Germany). Co-localization was analyzed using ZEN 3.0 software (Zeiss, Oberkochen, Germany) and ImageJ (National Institutes of Health, Bethesda, MD, USA).

### Western blot analysis

Renal cortex tissue was lysed in radioimmunoprecipitation assay (RIPA) buffer (Cat#R0278; Sigma-Aldrich, St. Louis, MO, USA) supplemented with a protease/phosphatase inhibitor cocktail (Cat#P3100-010; NCM Biotech, Suzhou, Jiangsu, China). Total protein concentrations were determined using a bicinchoninic acid (BCA) protein quantification kit (Cat#P0012S; Beyotime Biotechnology, Shanghai, China). Equal amounts of protein (30 µg per lane) were separated by 6–10% SDS-PAGE and transferred to polyvinylidene difluoride (PVDF) membranes (Millipore, Burlington, MA, USA).

Membranes were blocked with 5% non-fat dry milk (BD Biosciences, San Jose, CA, USA) for 1 h at room temperature and incubated overnight at 4 °C with the following primary antibodies: complement C3 (1:500, sc-28294; Santa Cruz Biotechnology, Dallas, TX, USA), C3aR (1:500, sc-133172; Santa Cruz Biotechnology, Dallas, TX, USA), Fibronectin (1:1,000, ab24130; Abcam, Cambridge, UK), osteopontin (1:1,000, sc-21742; Santa Cruz Biotechnology, Dallas, TX, USA), and NFATc1 (1:500, sc-7294; Santa Cruz Biotechnology, Dallas, TX, USA).

After washing, membranes were incubated for 1 h at room temperature with horseradish peroxidase (HRP)-conjugated secondary antibodies: goat anti-rabbit IgG (1:2,000, Cat#7074S; Cell Signaling Technology, Danvers, MA, USA) or goat anti-mouse IgG (1:2,000, Cat#7076S; Cell Signaling Technology, Danvers, MA, USA). Immunoreactive bands were detected with an enhanced chemiluminescence (ECL) kit (Cat#SQ201; EpiZyme, Shanghai, China) and visualized using a chemiluminescence imaging system (Model# 5200; Tanon Science & Technology, Shanghai, China).

To confirm equal protein loading, membranes were stripped with Restore™ PLUS Western Blot Stripping Buffer (Cat#46430; Thermo Fisher Scientific, Waltham, MA, USA) and reprobed with β-actin (1:2,000, Cat#sc-47778; Santa Cruz Biotechnology, Dallas, TX, USA), β-Tubulin (1:1,000, Cat#sc-5274; Santa Cruz Biotechnology, Dallas, TX, USA), or glyceraldehyde-3-phosphate dehydrogenase (GAPDH, 1:1000,Cat#ab181602; Abcam, Cambridge, UK).

Bound loading control antibodies were detected using species-matched HRP-conjugated secondary antibodies and ECL.

Band intensities were quantified by densitometry in ImageJ software, normalized to the respective loading control, and expressed as fold-change relative to the control group.

### Statistical analysis

All data were analyzed with GraphPad Prism software. Normality was assessed with the Shapiro-Wilk test. Results are presented as mean ± SD for normally distributed data or median (interquartile range) for non-normally distributed data.

For intergroup comparisons, non-normally distributed variables were analyzed by the Kruskal-Wallis test with Dunn’s *post-hoc* test. Normally distributed variables were assessed using the Brown-Forsythe test for variance homogeneity. If equal variances were confirmed, one-way ANOVA with Tukey’s HSD test *post-hoc* was used; if variances were unequal, Welch’s ANOVA followed by the Games-Howell test was applied. For time-course parameters, two-way ANOVA was performed, and specific group differences were evaluated using two-tailed unpaired Student’s t-tests. A *p*-value < 0.05 was considered statistically significant.

## Results

### Increased expression of complement C3 and C3a receptor (C3aR) in db/db mice

**Figure 1:**
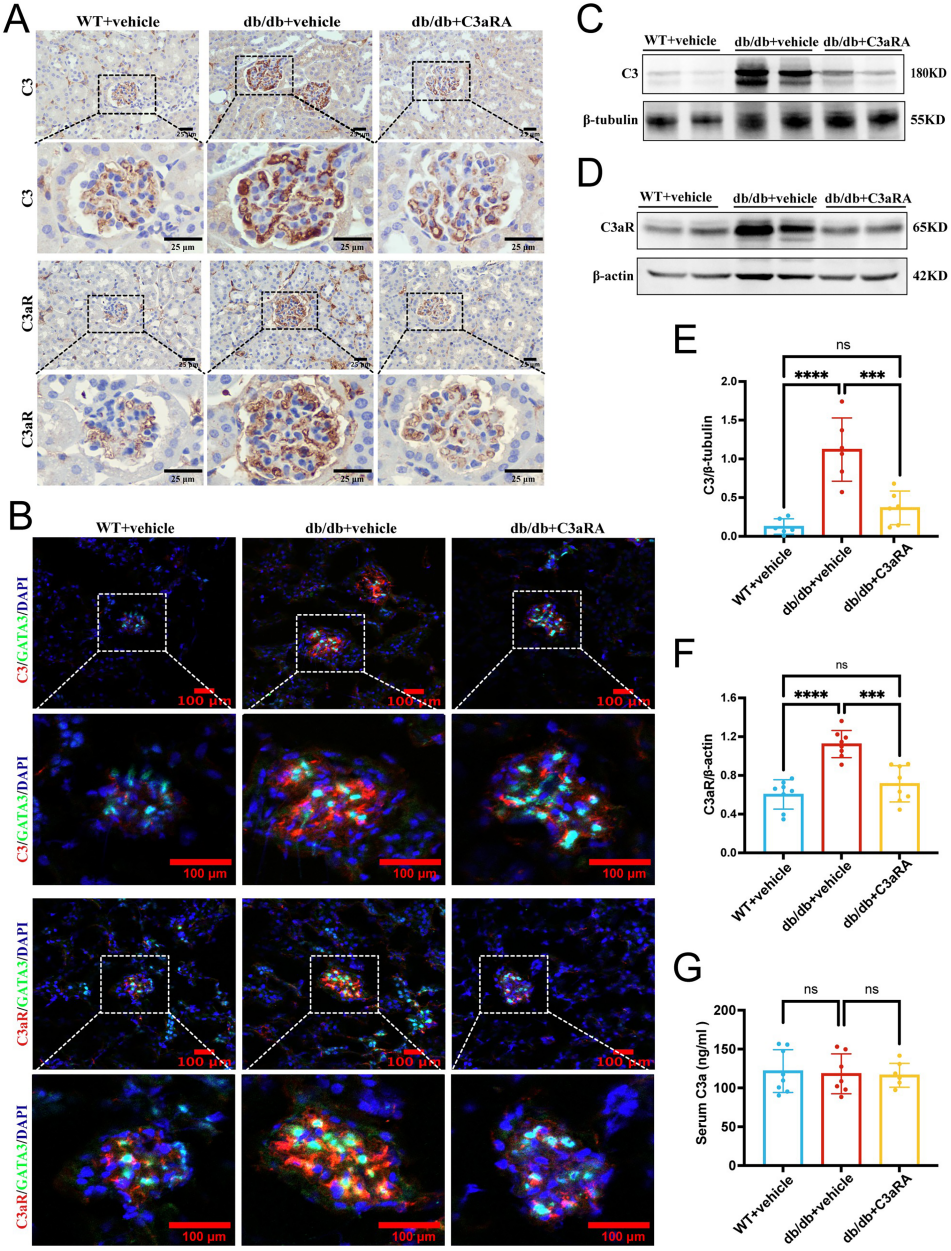
Expression and localization of C3 and C3aR in glomeruli of db/db mice and effects of C3aR antagonism. (A) Representative immunohistochemical staining of C3 and C3aR in each group (scale bar: 25 μm). (B) Immunofluorescence staining showing co-localization of C3 (red) or C3aR (red) with the mesangial cell nuclear marker GATA3 (green); nuclei counterstained with DAPI (blue); yellow signals in merged images indicate co-localization. Insets (4× magnification of boxed regions) display high-resolution details of C3/C3aR staining surrounding GATA3-positive mesangial nuclei, confirming mesangial compartment-specific co-localization. (scale bar: 100 μm (main images); 50 μm (insets)). (C, D) Representative Western blots of C3 and C3aR. (E, F) Quantitative analysis of C3 and C3aR protein expression (*n* = 6–8 per group). (G) Serum C3a levels in WT+vehicle (*n* = 8), db/db+vehicle (*n* = 7), and db/db+C3aRA (*n* = 8) groups. Data are expressed as mean ± standard deviation (SD). Statistical analyses were performed using one-way analysis of variance (ANOVA) with Tukey’s *post hoc* test or unpaired t-test. ****p < 0.0001; ***p < 0.001; ns, not significant.

To assess the role of complement in diabetic nephropathy, we compared the expression of C3 and C3aR in db/db mice and age-matched WT mice. Strong staining of C3 and C3aR was observed in the glomerular tufts of db/db mice, whereas expression was minimal in WT controls (Fig. 1A). Immunofluorescence confirmed that C3 and C3aR co-localized with GATA3, a nuclear marker of mesangial cells, suggesting a mesangial cell-specific role of C3aR in db/db mice (Fig. 1B). Treatment with the C3aR antagonist SB290157 (C3aRA) significantly reduced glomerular deposition of both C3 and C3aR. Western blot analysis corroborated these findings, showing decreased C3 and C3aR expression following C3aRA treatment (Figs. 1C–1F). In contrast, serum C3a levels did not differ significantly among the three groups (Fig. 1G).

### Effects of C3aR antagonism on metabolic and biochemical parameters

**Figure 2:**
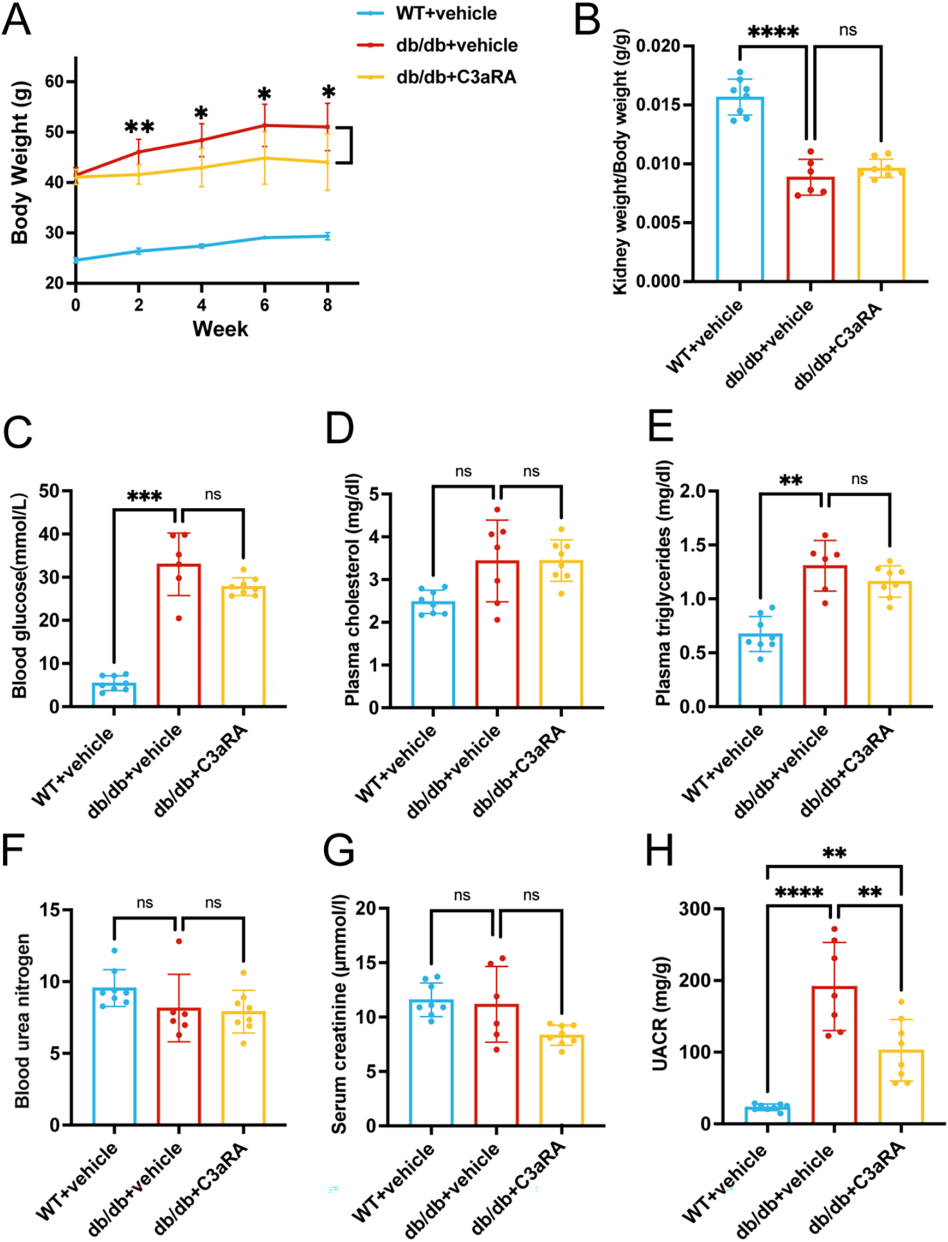
Effects of C3aR antagonism on metabolic and biochemical parameters in db/db mice. (A) Body weight changes over time (*n* = 8 per group). (B-H) Kidney weight-to-body weight ratio (B), blood glucose (C), plasma cholesterol (D), plasma triglycerides (E), blood urea nitrogen (F), serum creatinine (G), and urinary albumin-to-creatinine ratio at study endpoint (H) (*n* = 5–8 per group). Data are presented as mean ± SD. Two-way ANOVA with unpaired Student’s t-test was used for body weight comparisons; one-way ANOVA with Tukey’s *post hoc* test, Welch’s ANOVA with Games-Howell test, or unpaired t-test were applied for other parameters. *p < 0.05; **p < 0.01; ***p < 0.001; ****p < 0.0001 ns, not significant.

We next evaluated the impact of C3aR on metabolic parameters. At baseline and throughout follow-up, db/db+vehicle mice had significantly higher body weight than WT mice, consistent with a typical obese diabetic phenotype. Although db/db+C3aRA and db/db+vehicle mice exhibited similar body weights at baseline, C3aRA treatment moderated the rate of body weight gain in db/db mice over time (Fig. 2A). The kidney weight-to-body weight ratio was significantly lower in db/db+vehicle mice than in WT+vehicle mice. This ratio was partially restored by C3aRA treatment, but no significant difference was observed between db/db+vehicle and db/db+C3aRA groups at study completion (Fig. 2B).

As expected, db/db+vehicle mice displayed elevated blood glucose and plasma triglycerides compared to WT controls, with no significant differences in plasma cholesterol. C3aRA treatment did not alter these metabolic indices (Figs. 2C–2E). Regarding renal function, serum creatinine and blood urea nitrogen remained similar across groups (Figs. 2F, 2G). However, urinary albumin-to-creatinine ratio (UACR) was significantly higher in db/db+vehicle mice and was markedly reduced following C3aRA treatment (Fig. 2H).

### C3aR antagonism improves renal morphology

**Figure 3:**
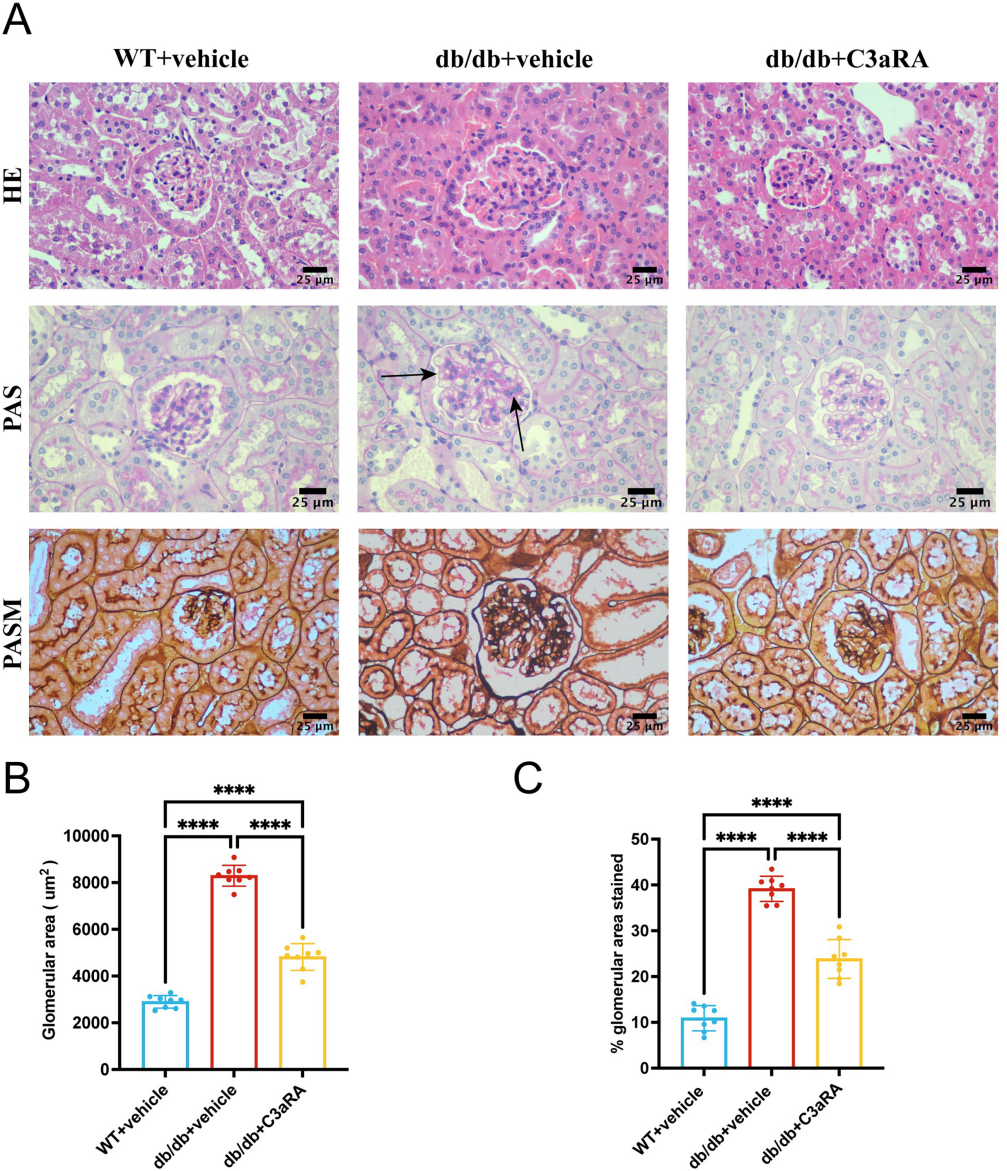
Renal histopathology in db/db mice and effects of C3aR antagonism. (A) Representative H&E, PAS, and PASM-stained kidney sections from each group (scale bar: 25 μm). (B) Quantification of glomerular area. (C) Percentage of glomerular area positive for PAS staining. Data are presented as mean ± SD (*n* = 8 per group). Statistical significance was assessed using one-way ANOVA with Tukey’s *post hoc* test or unpaired t-test; ****p < 0.0001.

Histological examination revealed pronounced renal pathology in db/db mice. HE staining demonstrated glomerular hypertrophy and an increase in mesangial cellularity, while PAS and PASM staining confirmed marked mesangial matrix expansion (Fig. 3A). These abnormalities were significantly ameliorated by C3aRA treatment. Quantitative analysis showed that both glomerular area and mesangial expansion were significantly increased in db/db mice compared with WT, and both were reduced by C3aRA treatment (*p* < 0.0001 for both; Figs. 3B, 3C).

### C3aR antagonism alleviates renal fibrosis

**Figure 4:**
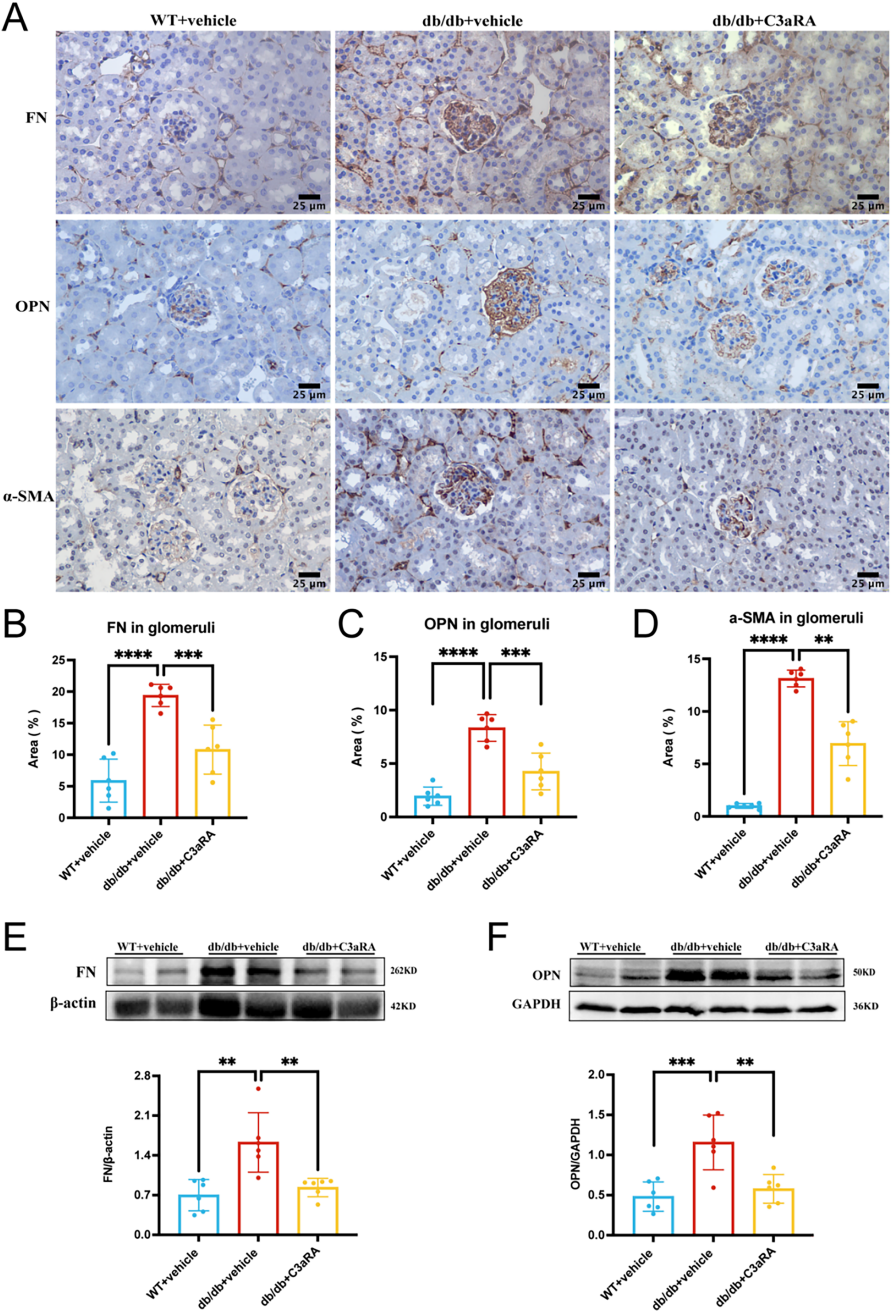
Expression of FN, OPN, and α-SMA in kidney tissues of db/db mice. (A) Immunohistochemical staining of FN, OPN, and α-SMA in each group (scale bar: 25 μm). (B–D) Quantitative analysis of FN (B), OPN (C), and α-SMA (D) expression (*n* = 6 per group). (E, F) Representative Western blots and quantitative analysis of FN (E) and OPN (F) protein expression. **p < 0.01, ***p < 0.001, ****p < 0.0001.

We then examined markers of renal fibrosis. Immunohistochemistry demonstrated increased deposition of fibronectin, OPN, and α-SMA in db/db mice, all of which were markedly reduced by C3aRA treat (Figs. 4A–4D). Western blot analysis confirmed that fibronectin and OPN expression were significantly downregulated following C3aRA treatment (Figs. 4E, 4F).

### C3aR regulates the CaN/NFATc1 signaling pathway

**Figure 5:**
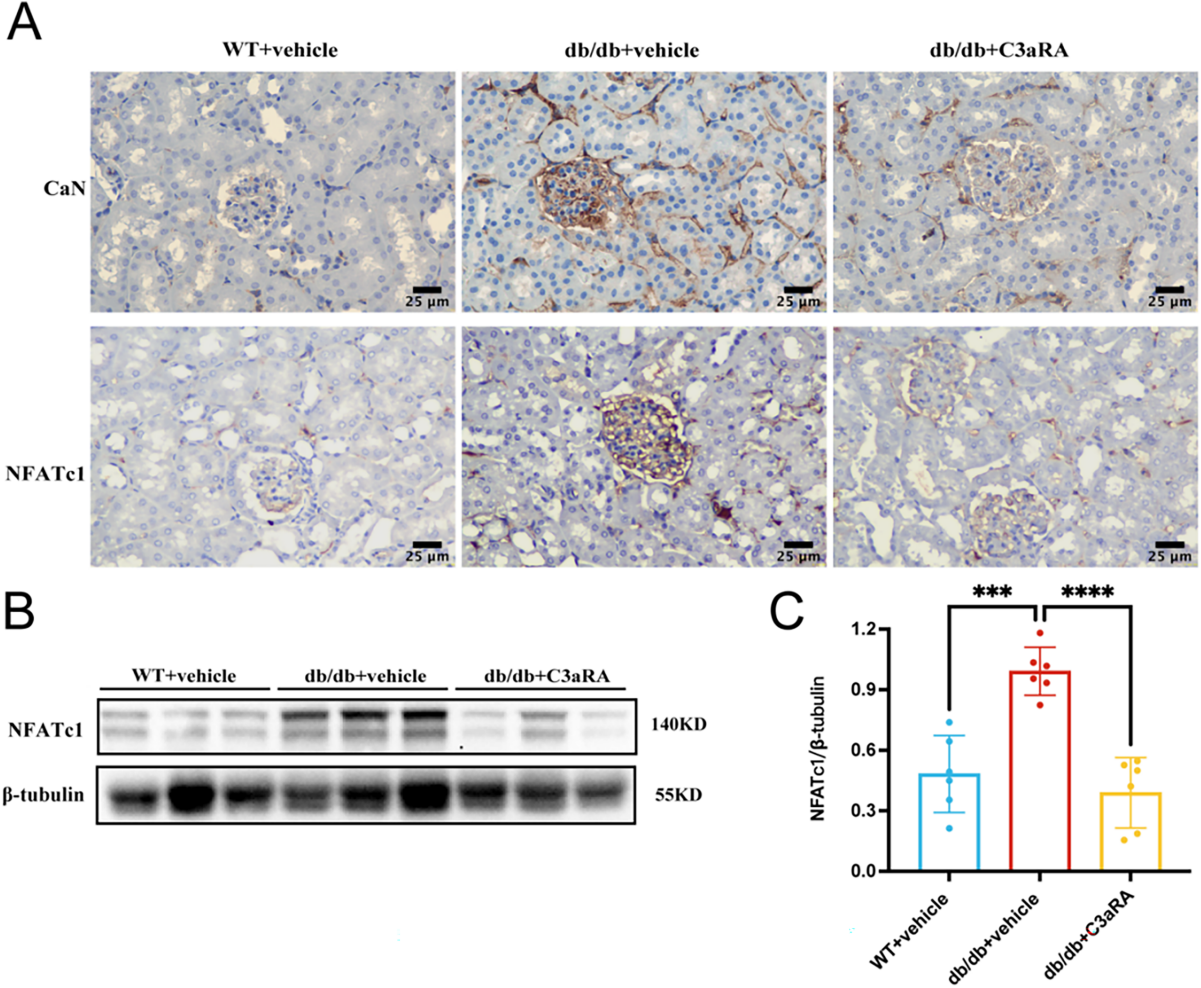
Expression of CaN and NFATc1 in kidney tissues of db/db mice. (A) Immunohistochemical staining for CaN and NFATc1 (scale bar: 25 μm). (B) Representative Western blot images of NFATc1. (C) Quantitative analysis of NFATc1 expression (*n* = 6 per group). ***p < 0.001, ****p < 0.0001.

To further explore the mechanism, we analyzed the CaN/NFAT pathway, a known regulator of mesangial cell activation. Immunostaining revealed increased CaN and NFATc1 deposition in the kidneys of db/db mice, which was significantly reduced following C3aRA treatment (Fig. 5A). Western blotting of kidney lysates confirmed decreased NFATc1 expression in the C3aRA group (Figs. 5B, 5C). Together, these results suggest that a potential association between C3aR signaling and the CaN/NFATc1 pathway in mesangial cell phenotypic and functional alterations.

## Discussion

Diabetic kidney disease (DKD) is the most prevalent and severe microvascular complication of DM and remains the leading cause of end-stage renal disease (ESRD), imposing a profound global health and socioeconomic burden (Tang et al., 2021). A hallmark feature of DKD is mesangial matrix accumulation, which manifests morphologically as diffuse or nodular mesangial expansion and thickening of the glomerular and tubular basement membranes (Akhtar et al., 2020).

The development of DKD involves a complex interplay of genetic predisposition, metabolic abnormalities, altered renal hemodynamics, and chronic inflammation (Barrera-Chimal & Jaisser, 2020). Among these, the complement system, an essential component of innate immunity, has recently attracted attention for its role in promoting immunoinflammation, progressive fibrosis, and chronic renal decline in DKD (Sun et al., 2022).

The complement system comprises more than 30 soluble and membrane-associated protein receptors, which, once activated, mediate enzymatic cascades central to host defense and inflammation (Noris & Remuzzi, 2013). Activation occurs *via* the classical, lectin, or alternative pathways, all converging on the cleavage of complement component C3. C3 is split by C3 convertases (C4b2a or C3bBb), triggering the downstream complement cascade and ultimately the formation of the membrane attack complex (MAC), which lyses pathogens and damaged cells (Ling & Murali, 2019). C3 cleavage generates two fragments, including the smaller anaphylatoxin C3a. C3a binds to its receptor, C3aR, a G-protein coupled receptor with seven transmembrane domains (Hawksworth et al., 2017). While C3a-C3aR signaling provides anti-infective and immune defense effects, excessive or uncontrolled activation drives proinflammatory signaling cascades, leading to cellular damage and disease progression (Gao, Cui & Zhao, 2020). SB290157, a non-peptide C3aR inhibitor identified by high-throughput screening, selectively blocks C3a binding to C3aR and prevents C3a-induced calcium mobilization and chemotaxis. Notably, this compound has well-documented pharmacological limitations, including potential off-target activity, weak agonist properties in certain cell types, and partial activation of C5aR2 (Lee, Taylor & Woodruff, 2020), which limits its use as a definitive tool for confirming C3aR function and supports its role as an exploratory tool for preliminary validation of C3aR-related pathways.

In this study, we used the C57BLKS/JGpt db/db mice mouse model, a well-characterized model of type 2 diabetic nephropathy that replicates key features of human DKD, including sustained hyperglycemia, glomerular hypertrophy, mesangial matrix expansion, and elevated UACR levels. Consistent with the human disease phenotype, we observed that db/db mice displayed markedly increased glomerular expression of C3 and C3aR, which correlated with mesangial matrix deposition and proteinuria. Selective inhibition of C3aR with SB290157 reduced the expression of matrix proteins FN, OPN, and α-SMA, suggesting that C3aR blockade alleviates mesangial matrix accumulation. In addition, C3aR inhibition appeared to attenuate activation of the CaN/NFATc1 signaling pathway. It should be noted that the present study was limited to male mice to avoid the confounding influence of the estrous cycle on metabolic and renal phenotypes; future studies will include both male and female animals to clarify potential sex-related differences in C3aR-mediated mesangial matrix expansion and diabetic kidney disease progression.

Clinical data also support this relationship. Serum and urinary C3a levels are significantly higher in diabetic patients with renal involvement compared to those without kidney damage (Huang et al., 2019). Urinary C3a strongly correlates with the severity of glomerulopathy, and systemic as well as local renal complement activation has been reported in patients with overt DKD (Zheng et al., 2019). Furthermore, renal tissue levels of C3a and C3aR increase progressively with DKD severity and correlate positively with glomerular injury score (GIS) and interstitial fibrosis/tubular atrophy score (IFTAS), both indicators of kidney injury (Morigi et al., 2020). These findings suggest that C3a/C3aR signaling is actively involved in DKD progression.

Our experimental data parallel these clinical observations. We detected localized complement activation in db/db mice, with C3 and C3aR co-localizing with the mesangial nuclear marker GATA3, confirming that mesangial cells in DKD aberrantly express C3aR. This is consistent with prior reports, as Braun et al. (2004) showed that mesangial cells in healthy kidneys lack C3aR expression, while Wan et al. (2007) demonstrated its upregulation under pathological conditions. Together, these findings imply that C3aR expression in mesangial cells is a disease-specific alteration contributing to pathology. Interestingly, there was no significant difference in serum C3a levels among our experimental groups, a result that diverges from human data. This discrepancy suggests that in db/db mice, C3aR activation is localized within the kidney rather than systemic.

Administration of the selective C3aR antagonist SB290157 did not lower circulating C3a levels but significantly downregulated C3 and C3aR expression in renal tissue. This pattern suggests that C3aR activation in the kidney may operate as a local positive feedback loop, amplifying complement-mediated injury during the course of DKD.

The tethered zone, located along the mid-axis of the glomerular capillary leaflets adjacent to the endothelium and basement membrane, is primarily composed of mesangial cells (MCs) and tethered stroma (Kurihara & Sakai, 2017). Mesangial matrix deposition is one of the earliest pathological features of DKD. As the disease advances, characteristic Kimmelstiel-Wilson nodules appear within the mesangial region, often accompanied by extensive tubulointerstitial lesions and eventual nephron loss in advanced stages (Thomas et al., 2015). In our db/db mouse model of early-stage diabetic kidney disease, serum creatinine and urea levels remained comparable across all groups, while the urinary albumin-to-creatinine ratio (UACR) was significantly elevated. This biochemical pattern indicates that albuminuria developed without a substantial reduction in estimated glomerular filtration rate (eGFR), implicating podocytopathy and glomerular filtration barrier dysfunction as the principal underlying mechanisms. The corresponding histopathology confirmed that the injury was localized to the glomerulus, characterized by hypertrophy and mesangial matrix expansion, without significant tubulointerstitial involvement, thereby identifying glomerular alterations as the key drivers of early albuminuria.

Mesangial cells are among the most metabolically active intrinsic cells of the glomerulus and are essential for constructing and maintaining the structural and functional homeostasis of the glomerular capillary network (Marciano, 2019). They synthesize and secrete stromal proteins, cytokines, and bioactive molecules, including inflammatory mediators, adhesion molecules, and chemokines. MCs also interact with endothelial cells and podocytes to preserve glomerular integrity (Zhao, 2019). Given their central role, MCs are major targets in both immune-mediated glomerular diseases, such as IgA nephropathy (Zhang et al., 2017), and metabolic disorders, such as diabetes mellitus (Avraham et al., 2021). Functional alterations in these tethered cells are therefore critical contributors to glomerular dysfunction during DKD progression.

Osteopontin (OPN) is significantly upregulated in the mesangial regions of diabetic kidneys (Nicholas et al., 2010). OPN interacts with ECM proteins such as FN and collagen, thereby facilitating mesangial matrix expansion (Lorenzen et al., 2008). In our study, intraperitoneal injection of SB290157, a selective C3aR antagonist, significantly reduced mesangial matrix proteins, including FN and OPN. Moreover, levels of α-SMA, a marker of MC transition to a myofibroblast-like phenotype, were also reduced. These findings strongly implicate local C3aR activation in mesangial matrix accumulation, consistent with earlier reports by Wan et al. (2007) and Li et al. (2014). Collectively, the evidence indicates that selective C3aR antagonism can mitigate mesangial matrix expansion in DKD.

To further investigate the mechanism by which C3aR promotes phenotypic and functional alterations in mesangial cells, we examined the CaN/NFATc1 signaling pathway. This pathway is regulated by Ca^2+^ release following activation of G-protein-coupled receptors, including C3aR. NFATc1, a key transcription factor within the NFAT family, is tightly controlled by Ca^2+^/CaN signaling. Under resting conditions, NFAT proteins remain phosphorylated in the cytoplasm. Upon ligand binding to surface receptors, phospholipase Cγ (PLCγ) is activated, hydrolyzing phosphatidylinositol bisphosphate (PIP_2_) into diacylglycerol (DAG) and inositol trisphosphate (IP_3_) (Hogan et al., 2003; Zhao et al., 2010). IP_3_ binds to its receptor (IP_3_R) on the endoplasmic reticulum (ER), triggering Ca^2+^ release into the cytoplasm. Elevated cystolic Ca^2+^ activates CaN, which dephosphorylates NFAT, enabling its nuclear translocation. Once in the nucleus, NFAT binds to promoters of target genes and regulates transcription (Gwack et al., 2007).

The CaN/NFATc1 pathway has been implicated in maintaining mesangial cells’ structure and function in kidney diseases (Cobbs & Gooch, 2007; Gooch et al., 2003). Importantly, CaN inhibitors such as cyclosporine (CsA) and tacrolimus (FK506) are widely used immunosuppressants in renal disorders, including lupus nephritis (Ponticelli, Reggiani & Moroni, 2021), membranous nephropathy (von Groote et al., 2021), and IgA nephropathy (Song et al., 2017).

Consistent with prior strategies that infer C3aR activation using downstream signaling and functional markers in models of diabetic nephropathy (Li et al., 2014), hypertensive kidney injury (Wang et al., 2023), and podocyte injury (Morigi et al., 2020), our study demonstrated elevated C3aR protein expression in the glomeruli of db/db mice. Importantly, antagonism of C3aR reduced CaN/NFATc1 expression, and these changes occurred in parallel with reductions in matrix proteins FN, OPN, and α-SMA. Together, these findings suggest that the CaN/NFATc1 pathway may regulate the expression of mesangial matrix proteins in diabetic nephropathy.

Despite these insights, our study has several limitations. First, protein quantification was performed on renal cortex tissue, which includes contributions from both tubules and surrounding interstitial cells. However, immunofluorescence and immunohistochemical analyses indicated that the observed protein expression differences were primarily localized to glomeruli. Future studies could address this limitation by employing laser capture microdissection (LCM) to specifically isolate glomeruli for mRNA and protein analysis, thereby distinguishing local protein synthesis from systemic deposition.

Second, while our findings were based on *in vivo* experiments, we did not perform complementary *in vitro* assays. Future cellular studies could apply C3aR agonists and antagonists to mesangial cells to directly assess the expression of matrix proteins and CaN/NFATc1 signaling molecules. Additional experiments using NFAT inhibitors such as 11R-VIVIT (NFAT2 inhibitor) could help clarify whether FN, OPN, and α-SMA expression is specifically mediated by NFAT signaling.

Finally, although SB290157 is widely used as a C3aR antagonist, it has inherent limitations in its pharmacological profile. Consistent with the IUPHAR/BPS guidelines (Harding et al., 2026), SB290157 was used as an exploratory tool for preliminary validation of the C3aR pathway in our diabetic kidney disease model, rather than a definitive tool for confirming C3aR function. Thus, our results do not establish C3aR as a definitive mediator, but rather identify C3aR as a candidate mediator of mesangial cell phenotypic transformation and mesangial matrix deposition. While our data suggest a potential association between C3aR signaling and the CaN/NFATc1 pathway in this process, the precise mechanisms remain to be fully understood. Key unanswered questions include how C3aR signaling modulates CaN/NFATc1 in mesangial cells, how this signaling axis drives phenotypic alterations and stromal protein expression, and whether upstream or downstream regulatory factors are also involved. These relationships require further validation through orthogonal approaches, such as C3aR genetic knockout or knockdown, to complement our pharmacological findings.

In conclusion, our findings demonstrated that pharmacological targeting of C3aR with SB290157 attenuates mesangial matrix deposition and may modulate mesangial cell phenotype and function in db/db mice. This work provides preliminary evidence that targeting C3aR may represent a promising therapeutic strategy for DKD.

## Supplemental Information

10.7717/peerj.21248/supp-1 Supplemental Information 1 Raw data for Figure 1.

10.7717/peerj.21248/supp-2 Supplemental Information 2 Raw data for Figure 2.

10.7717/peerj.21248/supp-3 Supplemental Information 3 Raw data for Figure 3.

10.7717/peerj.21248/supp-4 Supplemental Information 4 Raw data for Figure 4.

10.7717/peerj.21248/supp-5 Supplemental Information 5 Raw data for Figure 5.

10.7717/peerj.21248/supp-6 Supplemental Information 6 Uncropped Gels/Blots.

10.7717/peerj.21248/supp-7 Supplemental Information 7 ARRIVE checklist.

10.7717/peerj.21248/supp-8 Supplemental Information 8 C3a-ELISA data. ELISA was used to detect serum C3a levels. The raw data, including the standard curve, absorbance values of each sample, and calculated concentrations, were applied to evaluate the alterations in serum C3a expression among different groups.

## Additional Information and Declarations

### Data Availability

The following information was supplied regarding data availability:

The raw data is available in the Supplemental Files.
