# Supplementary figures and images for: Pharmacological targeting of C3aR modulates mesangial matrix deposition in db/db mice
Source: PeerJ. 2026 Apr 28;14:e21248. doi: 10.7717/peerj.21248 (PMC13134546; doi:10.7717/peerj.21248)

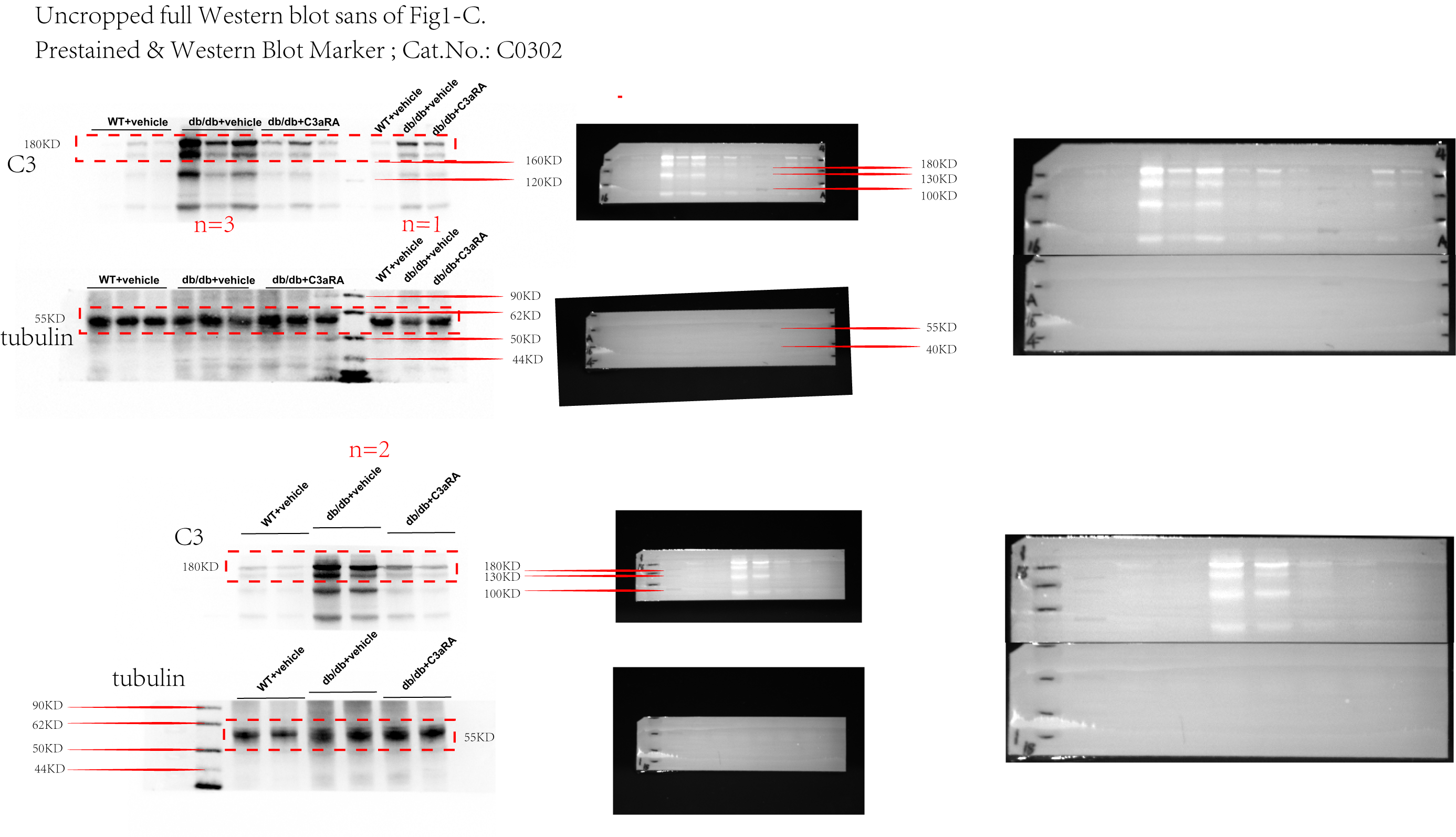

Supplement: Supplemental Information 6 [file peerj-14-21248-s006.zip › Western blot sans/C3-tubulin.tif]

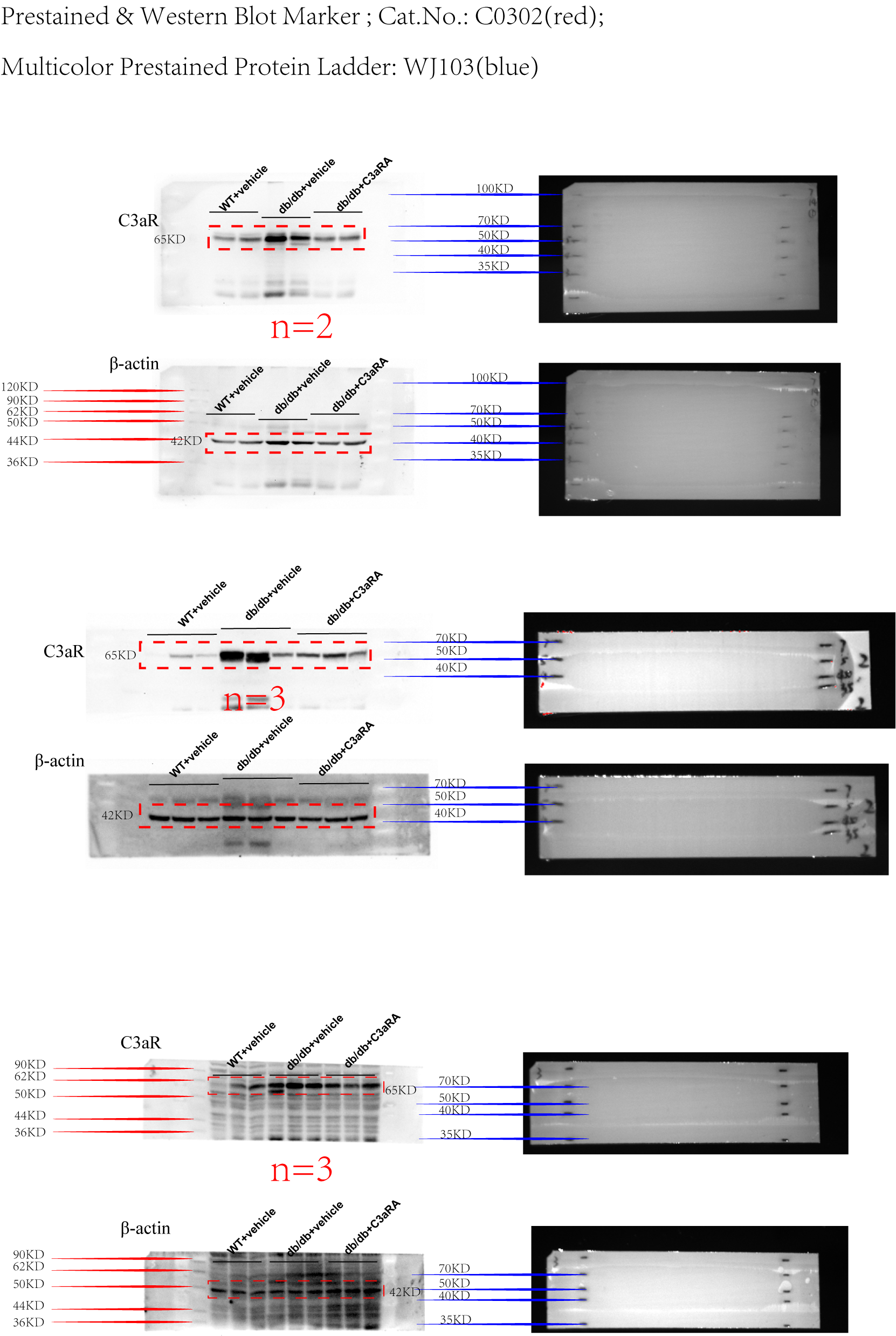

Supplement: Supplemental Information 6 [file peerj-14-21248-s006.zip › Western blot sans/C3aR-actin.tif]

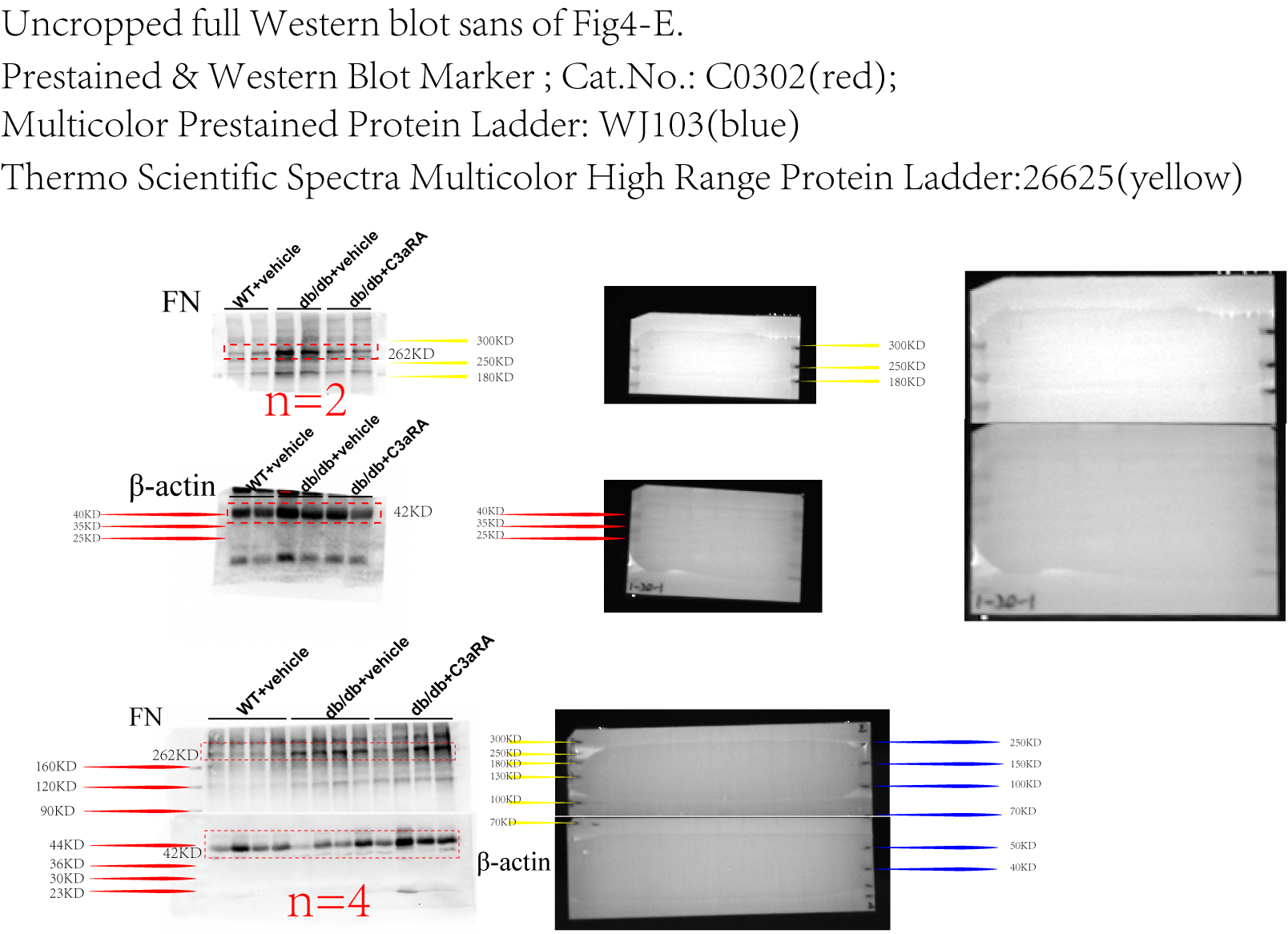

Supplement: Supplemental Information 6 [file peerj-14-21248-s006.zip › Western blot sans/fn-actin.tif]

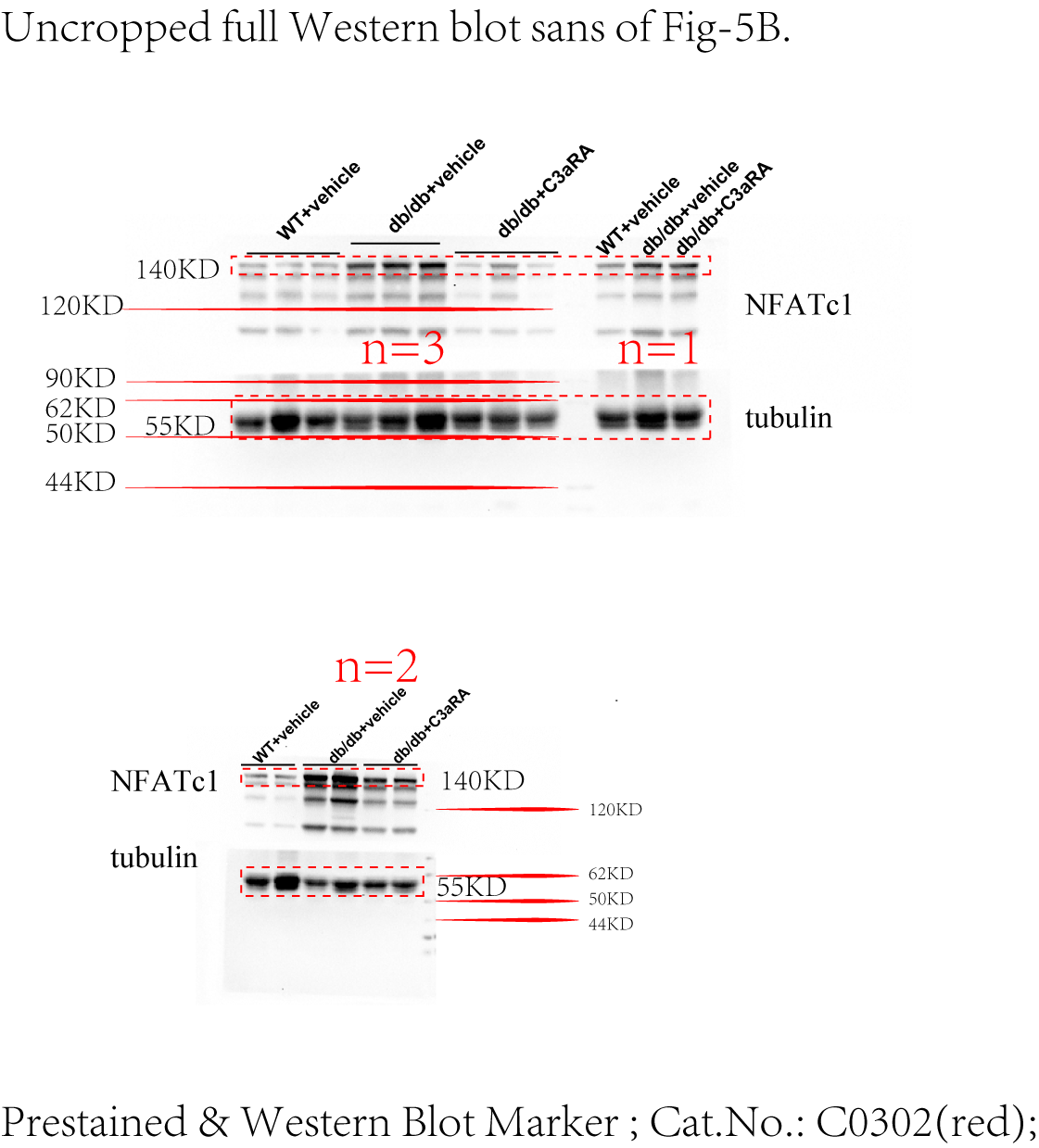

Supplement: Supplemental Information 6 [file peerj-14-21248-s006.zip › Western blot sans/NFATc1-tubulin.tif]

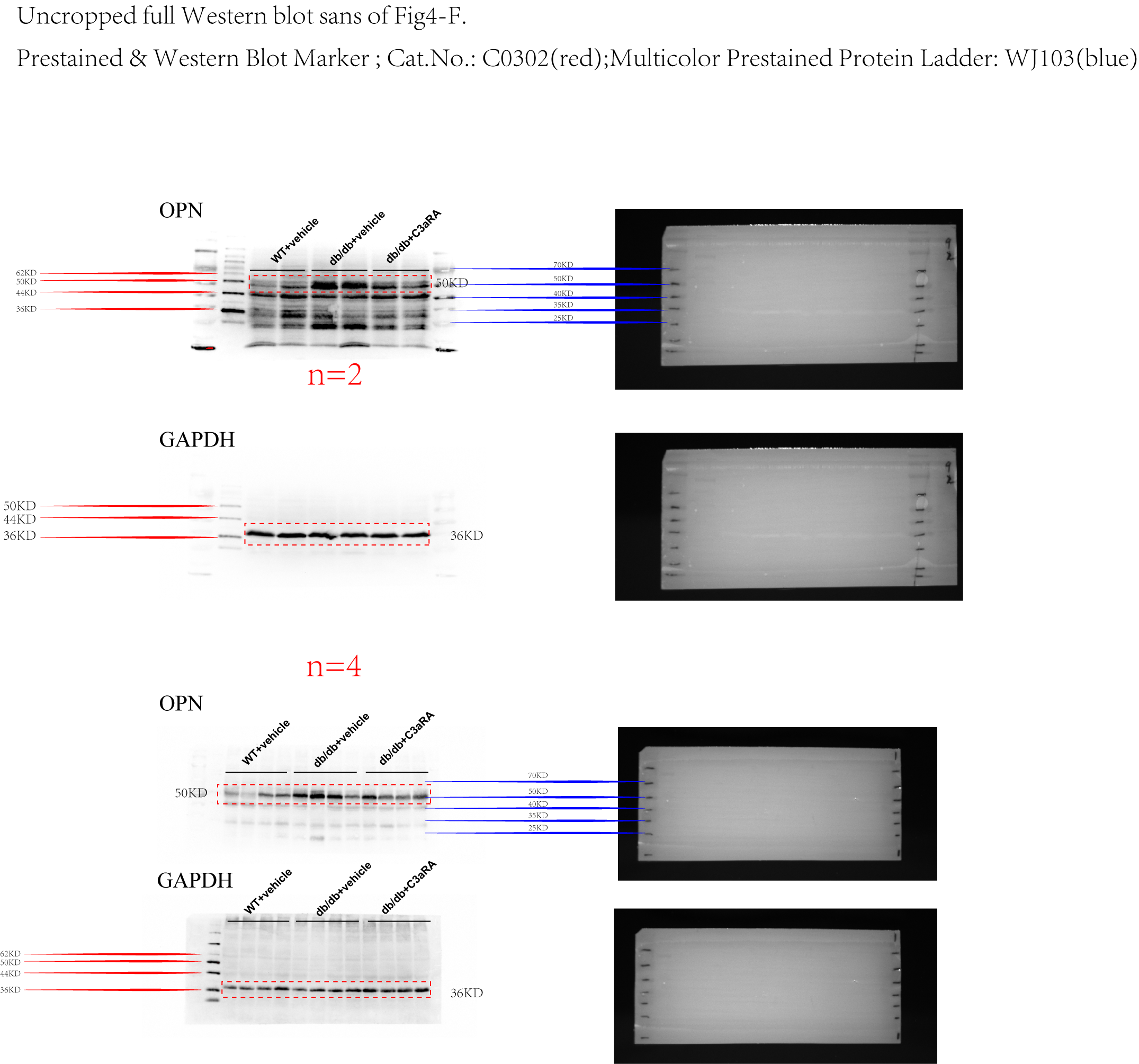

Supplement: Supplemental Information 6 [file peerj-14-21248-s006.zip › Western blot sans/opn-GAPDH.tif]
